# Resistance of Adult *Kryptolebias marmoratus* Hermaphrodites to Irreversible Sex Change by Exogenous Androgens

**DOI:** 10.1093/iob/obag009

**Published:** 2026-03-11

**Authors:** J Ficklin, S L Moy, A Sinha, E S Haag

**Affiliations:** Department Biological Sciences Graduate Program, University of Maryland, 4066 Campus Drive, College Park, MD 20742, USA; Department of Biology, University of Maryland, 4066 Campus Drive, College Park, MD 20742, USA; Department of Biology, University of Maryland, 4066 Campus Drive, College Park, MD 20742, USA; Department Biological Sciences Graduate Program, University of Maryland, 4066 Campus Drive, College Park, MD 20742, USA

## Abstract

The *Kryptolebias marmoratus* species complex contains the only known self-fertile hermaphroditic vertebrates. All three taxa in this clade live in mangrove forests of the Caribbean basin, and all have individuals with both testis and ovarian tissue in their gonads. Two, *K. marmoratus* and *K. hermaphroditus*, use self-fertility as their main mode of reproduction and have colonized remote islands. *K. marmoratus* also has well-documented production of fertile males through sex change of adult hermaphrodites. The control of sex change in *K. marmoratus* is poorly understood. Individuals believed to be genetically identical can be raised in the same environment, yet change sex at drastically different times or not at all. However, juvenile fish can be permanently masculinized by immersion in the androgen methyltestosterone (MT). Here, we first document substantial individual variation in overall gonad size and in testis content within and between the gonads of adult *K. marmoratus* hermaphrodites. This led us to hypothesize that variation in testis-derived androgen may create a positive feedback that drives sex change in some individuals, but not others. We used endocrine manipulations to test a prediction of this hypothesis, namely that sex change in adults could be triggered by exogenous androgens. While exposure to MT led to full masculinization of pigmentation and partial masculinization of the gonad, it was not enough to maintain a permanent transition like that seen in a natural sex change. Additional treatments with 11-ketotestosterone and the aromatase inhibitor fadrozole had weaker effects, which were also impermanent. In this way, adult *K. marmoratus* differ from larvae, and from adults of other gonochoric teleosts (i.e., with true females and males), in which permanent masculinization of females has been reported after administration of both androgens and aromatase inhibitor. This resistance may reflect changes in adult sexual physiology that evolved to address the underlying need of hermaphrodites to maintain many female traits despite the presence of elevated androgens in a common circulatory system.

## Resumen

El complejo de especies *Kryptolebias marmoratus* contiene los únicos vertebrados hermafroditas autofértiles conocidos. Los tres taxones de este clado viven en los manglares de la cuenca del Caribe y todos presentan individuos con tejido testicular y ovárico en sus gónadas. Dos de ellos, *K. marmoratus* y *K. hermaphroditus*, utilizan la autofertilidad como su principal modo de reproducción y han colonizado islas remotas. *K. marmoratus* también presenta una producción bien documentada de machos fértiles mediante el cambio de sexo de hermafroditas adultos. El control del cambio de sexo en *K. marmoratus* es poco conocido. Individuos considerados genéticamente idénticos pueden criarse en el mismo entorno, pero cambiar de sexo en momentos drásticamente diferentes o no hacerlo en absoluto. Sin embargo, los peces juveniles pueden masculinizarse permanentemente mediante la inmersión en el andrógeno metiltestosterona. Aquí, documentamos primero una variación individual sustancial en el tamaño general de las gónadas y en el contenido testicular dentro y entre las gónadas de hermafroditas adultos de *K. marmoratus*. Esto nos llevó a plantear la hipótesis de que la variación en los andrógenos derivados de los testículos puede crear una retroalimentación positiva que impulsa el cambio de sexo en algunos individuos, pero no en otros. Utilizamos manipulaciones endocrinas para probar una predicción de esta hipótesis, a saber, que el cambio de sexo en adultos podría ser desencadenado por andrógenos exógenos. Si bien la exposición a la metiltestosterona (MT) condujo a la masculinización completa de la pigmentación y a la masculinización parcial de la gónada, no fue suficiente para mantener una transición permanente como la observada en el cambio de sexo natural. Los tratamientos adicionales con 11-cetotestosterona (KT) y el inhibidor de la aromatasa fadrozol tuvieron efectos más débiles, que también fueron impermanentes. De esta manera, los adultos de *K. marmoratus* difieren de las larvas y de los adultos de otros teleósteos gonocóricos (es decir, con machos y hembras verdaderas), en los que se ha descrito una masculinización permanente de las hembras tras la administración de andrógenos e inhibidores de la aromatasa. Esta resistencia podría reflejar cambios en la fisiología sexual adulta que evolucionaron para abordar la necesidad subyacente de los hermafroditas de mantener muchos rasgos femeninos a pesar de la presencia de niveles elevados de andrógenos en un sistema circulatorio común.

## Introduction

Hermaphroditic organisms exhibit female and male traits over the course of their life. Sequential hermaphrodites start their lives as one sex and, after some cue, can transition to the other. For example, the anemonefish, *Amphiprion*, begin life as males, but can become female (Iwata et al. 2008). The trigger for sex change can be social, environmental, or physiological (Avise 2011). In all cases, sex change modifies the gonads to replace the production of one gamete type with the other. When present, secondary sexual traits, such as sex-specific pigmentation or ornaments, are also converted. The order of sexes (i.e., protogyny or protandry) is shaped by size- and age-dependent influences on raw fecundity and the dynamics of sexual selection (Ghiselin 1969; Avise and Mank 2009; Kazancioglu and Alonzo 2010).

Simultaneous hermaphrodites can reproduce using both sperm and eggs within the same time period. Ovary and testis function may still be separated in time when sperm are stored, as in the nematode *Caenorhabditis elegans* (L’Hernault 2009), or united in a gonad that stably contains both ovarian and testicular tissue, as in the related nematode *Auanema rhodensis* (McCaig et al. 2017). Relevant to this study, simultaneously hermaphroditic teleost fishes follow the latter pattern (Qu et al. 2020; Kuwamura et al. 2022; Oyama et al. 2023).

A subset of simultaneous hermaphrodites is self-fertile. While selfing introduces extreme inbreeding, it also provides reproductive assurance at low density (Baker 1955). Over time, recessive deleterious alleles can be purged, leading to completely homozygous genotypes that are nevertheless healthy (Hartfield 2016). Within plants, selfing is relatively common, with the majority of temperate and roughly half of tropical hermaphrodites regularly selfing (Jarne and Charlesworth 1993). Among animals, most simultaneous hermaphrodites are marine invertebrates, but only about a third of these show signs of regular selfing (Jarne and Auld 2006). Though rare in some taxa, self-fertility is an asset for laboratory genetics research. Selfers are naturally resistant to inbreeding depression, and one founding heterozygote can produce homozygous offspring. For these reasons, *Arabidopsis thaliana* and *C. elegans* were chosen as model organisms over strictly outcrossing relatives in (Nigon 1949; Brenner 1974; Tang et al. 2007). In *A. thaliana*, selfing evolved through loss of an ancestral self-incompatibility system (Charlesworth and Vekemans 2005; Tang et al. 2007), whereas in *C. elegans* it required acquisition of a novel spermatogenic program by the otherwise female XX sex (Haag et al. 2018).

This study concerns the only known self-fertile vertebrate, the mangrove killifish *Kryptolebias marmoratus* (also called the mangrove rivulus, from its former membership in the genus *Rivulus*). *Kryptolebias marmoratus* is also notable (though not unique) for being a sequential hermaphrodite (Harrington 1961; Harrington 1971; Avise and Tatarenkov 2015). *Kryptolebias marmoratus* is found from the north coast of Brazil northward through Central America and the Caribbean, and as far north as southern Florida (Harrington 1961; Costa 2016). The hermaphrodite is drab with largely ovarian gonads, similar to females of related species, but produces small pockets of testis tissue along the oviduct (Harrington 1961; Cole and Noakes 1997; Kanamori et al. 2006; Qu et al. 2020). This enables internal self-fertilization. Hermaphrodites have not been observed to outcross with each other (Hee et al. 1998; Furness et al. 2015). However, the brightly pigmented males can fertilize the small proportion of nonselfed eggs released by hermaphrodites in both lab and field conditions, making *K. marmoratus* functionally androdioecious (Mackiewicz et al. 2006a, 2006b, 2006d). This is reminiscent of *C. elegans*, and due to the ease of laboratory culture *K. marmoratus* is an emerging model organism (Orlando 2012; Taylor 2012). Recent research has focused on phenotypic plasticity (Earley et al. 2012; Wells et al. 2015; Heffell et al. 2018), behavior (e.g., Li et al. 2014; Li et al. 2018; Carion et al. 2020; Fortunato and Earley 2023), genomics (Lee et al. 2008; Kelley et al. 2016), developmental genetics (Sucar et al. 2016; Saud et al. 2021; Li et al. 2023), and population genetics (e.g., Tatarenkov et al. 2007; Tatarenkov et al. 2010; Tatarenkov et al. 2012; Avise and Tatarenkov 2012; Tatarenkov et al. 2015). Here, we are concerned with the relationship (if any exists) between self-fertility and sex change.

In most inbred strains of *K. marmoratus*, only a minority of adult hermaphrodites change sex into males (Gresham et al. 2025). Sex change is not under obvious social control, as fish can (but mostly do not) change sex when reared in isolation. This suggests the trigger may be distinct from the neuroendocrine mechanisms that operate in other sequential hermaphrodites. However, the lifetime probability and average age of change can vary greatly, due at least in part to interstrain genetic variation (Turner et al. 1992; Grageda et al. 2005; Turner et al. 2006, Tatarenkov et al. 2015; Gresham et al. 2025). After transition, male gonads are entirely testis and their pigmentation and behavior become similar to that of males of gonochoric relatives (Scarsella et al. 2018).

The unique combination of both simultaneous and sequential hermaphroditism found in *K. marmoratus* poses a number of puzzles. Sexual development in vertebrates, including teleosts, is generally guided and reinforced by circulating gonadotropin (peptide) and steroid hormones (Baroiller et al. 1999; Guiguen et al. 2010). The latter signal through conserved androgen receptors (ARs) and estrogen receptors (ERs) that are expressed in the gonad, brain, and other tissues (Li et al. 2014; Seo et al. 2006). These previous studies mention a single homolog, but other teleosts generally have two paralogs (e.g., Olsson et al. 2005), ARα and ARβ.

In gonochoric fish, steroids have distinct profiles in males (higher levels of certain androgens) and females (more of certain estrogens (Brantley et al. 1993; Borg 1994). In both gonochoric and sex-changing teleosts, treatments that mimic the alternative or terminal sex’s hormone profile can trigger irreversible sex change (Frisch 2004; Godwin 2010). For example, adult females of the honeycomb grouper, a protogynous sequential hermaphrodite, can be pushed into premature maleness by the application of 11-keto testosterone (KT; Bhandari et al. 2006). KT is a potent agonist of ARs (Miura et al. 1991; Hossain et al. 2008), and in some protogynous teleosts is more tightly correlated with male phenotype than is testosterone (Nakamura et al. 1989; Bhandari et al. 2003). This is potentially relevant to *K. marmoratus* as well. Embryos immersed in the synthetic androgen 17-alpha-methyl testosterone (MT) shortly before hatching mature not as the usual hermaphrodite but as functional males (Kanamori et al. 2006). Further, adult hermaphrodites given a single injection of various concentrations of MT exhibit a transient reduction in oocyte production, with no apparent impact on testes (Park et al. 2013). However, while unmanipulated adult *K. marmoratus* hermaphrodites that are not undergoing sex change excrete less 11-ketotestosterone than do males (as expected), there is surprisingly no clear sex effect on secreted testosterone (Garcia et al. 2016).

In addition to direct application of exogenous sex steroids, pharmacological perturbation can also induce sex change. For example, adult females of the gonochoric Nile tilapia (*Oriochromis niloticus)* and zebrafish (*Danio rerio)* can be irreversibly transformed into males by the application of aromatase inhibitors, which prevent the formation of estrogens from androgen precursors (Takatsu et al. 2013; Sun et al. 2014). Aromatase inhibition can also masculinize females of the sequentially hermaphroditic blackeye (*Coryphopterous nicholsii*) and coral (*Gobiodon erythrospilus*) gobies (Kroon and Liley 2000; Kroon et al. 2005). This may be mimicking a natural process, as downregulation of gonadal aromatase expression is an early step in natural female-to-male sex change in the bluehead wrasse *(Thalassoma bifasciatum*; Todd et al. 2019). Brain aromatase is similarly downregulated in the bluebanded goby (*Lythrypnus dalli*) (Black et al. 2005). While these results have been interpreted as evidence that estrogen inhibits sex change to male, accumulation of the androgens that are aromatase’s substrate likely plays a role in some cases: post-fadrozole increase in plasma testosterone has been observed in taxa as diverse as birds and teleost fish (Soma et al. 2000; Ankley et al. 2002; Huffman et al. 2013).

Here, we attempt to address several related questions about the sexual biology of *K. marmoratus.* As the adult hermaphrodite stably maintains ovarian and testicular gonad tissues, what breaks this balance to allow transition in older adults? When it does occur, how are ovarian regression and testis proliferation coordinated? Since effectively clonal hermaphrodites do not all change sex, what determines which individuals will change, and which will not?

We first sought to characterize the extent of variation in sex allocation within outwardly similar hermaphrodites of the same inbred lines. We find substantial within- and between-individual variation in gonad size and testis content among similarly sized individuals. Given that steroid hormones can drive sex change in other protogynous fish, and that the somatic testis is a major source of androgen production (Tokarz et al. 2015), and the variable age at which clonal hermaphrodites undergo sex change in *K. marmoratus* (Harrington 1971), we hypothesized that hermaphrodites that change sex are those with a larger initial testis endowment. This may set up a positive feedback that carries androgen levels above a crucial threshold that triggers sex change. We term this the “androgen snowball hypothesis.” Those that remain below the putative threshold would die as terminal hermaphrodites. Alternatively, because they maintain mosaic male and female gonads for long periods, sex change in adults *K. marmoratus* hermaphrodites may instead rely on an unknown trigger.

We sought to test these alternative hypotheses with chronic, masculinizing perturbations, including one that leads to direct male development of juvenile *K. marmoratus*. This was particular appealing because successful induction of sex change would facilitate analyses of the transition process, which is currently difficult due to the unpredictability of its initiation. Alternatively, if we observed either no effect or a reversible effect on sex, we would conclude that hermaphrodites rely on a nonhormonal trigger of sex change. This could reflect a classic organization-activation dynamic (Phoenix et al. 1959; Arnold and Breedlove 1985), a developmental progression in which early, “organization” processes are sensitive to sex reversal but once completed establish “activation” systems that are intrinsically more resistant. However, as numerous other teleosts can be permanently sex-reversed in adulthood by endocrine manipulation, the inability to do so may indicate derived aspects of *K. marmoratus’* sexual physiology.

In our manipulations, we employed MT because of its ability to sex-reverse embryos (Kanamori et al. 2006) and its impact on hermaphrodite oogenesis (Park et al. 2013). We administered KT because of its known role in female-to-male sex change in other sequentially hermaphroditic fish. We also treated hermaphrodites with fadrozole, a nonsteroid drug. Fadrozole was first shown to be a potent inhibitor of estrogen biosynthesis in mammals (Steele et al. 1987; Santen et al. 1991), and later found to have similar activity in gonochoric teleost fish (e.*g.*, Ankley et al. 2002).

## Methods

### AR phylogeny

The longest predicted protein product from each unique annotated AR gene was obtained from ENSEMBL (www.ensembl.org) for *K. marmoratus* (ENSKMAG00000016405, ENSKMAG00000004997), *O. latipes* (ENSORLG00000009520, ENSORLG00000008220), and from NCBI Genbank for *F. heteroclitus* (XP_012720156, XP_012725354). Protein products were aligned in Geneious software, and a neighbor-joining phylogeny was generated using default parameters.

### Fish care

*Kryptolebias marmoratus* of SOB-8 and SOB-10 inbred strains were originally collected in the Florida Keys (Mesak et al. 2015) and were the kind gift of Dr. Ryan Earley. Animals were raised individually in 750 mL of brackish water in 1 L food-grade polypropylene tanks (Rubbermaid). Water was prepared with centrally supplied deionized water supplemented with Instant Ocean Sea Salt at 12.5ppt. Light cycles were set at 12 h on and 12 h off, with water temperatures maintained between 26.5 and 28.5°C. Fish were fed 6 days out of the week with *Artemia* brine shrimp nauplii, amounting to those emerging from 0.1 mL dry cysts. Hatched nauplii were separated from hatching medium after 48 h (35 PPT sea salts) with a fine mesh net, and resuspended in roughly 5–10 PPT water before feeding to reduce salt accumulation. Fish were monitored daily for health and signs of external morphological changes. All husbandry procedures and animal protocols were approved by our Institutional Animal Care and Use Committee (IACUC).

### Hormone manipulations

Hermaphroditic adults were judged by the presence of posterior ocellus, ongoing oviposition, and lack of orange pigmentation or ventral tail bar. Animals of 2 years of age or older were selected, as these are almost always “terminal hermaphrodites” that are unlikely to change sex naturally. Their tanks were moved to a common shelf, isolated from the rest of the colony (though in the same room). In previous studies, exogenous hormones have been administered to fish via injection, feed, or simple immersion. We chose the latter for its simplicity and efficacy that is comparable to feeding in other species (e.g., Takahashi 1975; Wassermann and Afonso 2003; Luzio et al. 2015; Lee et al. 2017; Suseno et al. 2020).

Long-term stocks of hormones were made in DMSO at 100 mg/mL and stored at –20°C until use. Working stocks (0.4 mg/mL) were made by diluting 0.2 mL DMSO stocks in 50 mL fish culture water. After a complete water change, the water in their tanks was dosed with working stock solutions to final tank concentrations of either 100 ng/mL or 50 ng/mL for MT, 100 ng/mL for KT, and 5 ng/mL for fadrozole. MT doses for immersion were chosen to be two- and four-fold higher than those used to sex-reverse near-hatching larvae by Kanamori et al. (2006). The chosen KT dose was the same used by Takahashi (1975), while that for fadrozole was modeled after Schroeder et al. (2017). Hormone stocks were purchased from Sigma Chemical (for MT under DEA Registration RH0521155), and dissolved in DMSO at 100 mg/mL and stored frozen (–20°C). Just prior to use, stocks were diluted in culture water to 0.4 mg/mL and the appropriate amount was added to 750 mL tanks. Because the rate of hormone inactivation in the water was unknown and complete changes of water can be stressful, we implemented a partial replacement scheme: Every 10 days, half the tank’s water was removed and replaced with fresh aquarium water and re-dosed to bring the concentration of the hormones back to their starting concentrations, assuming all active hormones were metabolized or otherwise broken down. If instead hormone persisted with no loss, the day 10 change would elevate the hormone concentration to 1.5 times the target dose, and the second (day 20) to 1.75 times the target. The likely dose is between these two extremes, but it was not measured.

A total of 50 fish were used, divided into five treatment groups: 100 ng/mL MT (*n* = 13), 50 ng/mL MT (*n* = 13), 100 ng/mL KT (*n* = 13), 5 ng/mL fadrozole (*n* = 6), and control (*n* = 5). On day 1 the fish were placed into the various treatments, and drug exposure was maintained as described in the previous paragraph. On day 30, all fish were removed from the hormone-treated water and returned to fresh aquarium water lacking drugs. All control and fadrozole-treated fish, four 50 ng/mL MT fish, four 100 ng/mL MT fish, and eight KT fish were immediately sacrificed to examine early effects (Fig. 6). The remaining androgen-treated fish were left to be observed for up to 60 days posttreatment (Fig. 5). Untreated control hermaphrodites were chosen using the same criteria used for experimental animals and experienced water changes and colony positions identical to hormone-treated fish, with the exception that no DMSO vehicle was added.

### Hormone immunoassays

While fadrozole can reduce estrogen and increase androgen titers in gonochoric teleost fish (e.g., Ankley et al. 2002), its net effect on sex steroids has not been characterized in *K. marmoratus*. We therefore obtained hormonal profiles of our fadrozole-treated fish, along with control fish whose gonads were quantified for overall size and testis content (Fig. S1). The hormonal assay procedure is based on previous noninvasive assay methods used in *K. marmoratus* and other species (Scott et al. 2008; Earley et al. 2013; Garcia et al. 2016; Scarsella et al. 2016; Houslay et al. 2019; Houslay et al. 2022). Fish treated with fadrozole were placed in 500 mL of clean water for 30 min immediately following the end of their 30-day exposure. After 30 min the fish were returned to their prior containers, now without the fadrozole. The water in which they were incubated was filtered using Whatman grade 1 filter paper to remove large particulates, and then adjusted to pH 3 using formic acid.

A Waters C18 500 mg solid-phase extraction column was primed for each fish by flushing it with two washes of 2 mL HPLC grade-methanol followed by two washes of 2 mL of ultrapure water. The hormones were then extracted from the water samples using a vacuum pump to pass them through the column. The columns were then purged with a 2 mL wash of ultrapure water to remove extra salt. Hormones were then eluted with two washes of 2 mL of HPLC-grade ethyl acetate. The elution solvent was evaporated at 37°C with a gentle stream of nitrogen gas passing over the samples through an evaporation manifold. The hormone-containing residues were resuspended using 50 µL of the proprietary buffers provided in the Cayman Chemical ELISA kits for testosterone (#582701), KT (#582751), and estradiol (#501890) following the protocol provided in said kits. Hormone levels were measured in a BioTek Epoch plate reader via a regression compared to standard curves based on serial two-fold dilutions (see Fig. S2). The numbers reported in Fig. Fig. 6 represent concentrations in the final suspensions. Assuming 100% efficient isolation, the concentration of hormone released into the 500 mL of assay water in 30 min is 1/10,000 of the value reported.

### Histology

Fish were euthanized with 0.2% bicarbonate-buffered tricaine (MS222) in culture water, followed by decapitation. The visceral masses of the fish, including gonads and intestines, were immediately fixed using Bouin’s fixative for 24 h, followed by a 24 h bath of 70% ethanol to remove excess fixative. The tissue was then exposed to increasing concentrations of ethanol up to 100%, and cleared with Histoclear II (Electron Microcopy Sciences). The tissue was embedded in paraffin, cross-sectioned at 8 µm or 10 µm thickness, and the sections were bound to positively charged slides (Fisher Permafrost Plus) using a slide warmer. Slides were stained using hematoxylin and eosin (Humason 1979).

### Image analysis

Stained sections were photographed under 3.15× magnification using a Zeiss AxioCam digital camera and Zen Blue microscope imaging software. If the gonad were larger than the view field, then multiple images would be composited together using the Zen Blue live panorama function to get a complete image of the tissue. The total cross-sectional area of the section as well as the area of testis, oocytes, oviduct, and amorphous/discolored tissue were measured using the Freehand Selection Tool in ImageJ (Schneider et al. 2012). The testis was diagnosed by the presence of densely nucleated tissues with little cytoplasm along the edge of the oviduct (appearing bright indigo in aggregate when stained with hematoxylin). Oocytes were recognized as being much larger ovoid cells with large nuclei and web-like cytoplasm or (at the oldest stages) yolk aggregates. Amorphous and discolored tissue was diagnosed by the presence of melanic pigment and/or signs of oocyte degradation and lack of defined cellular structures. Such tissue was not seen in untreated hermaphroditic ovotestes or in male testes. The oviduct, an open tube that runs laterally down the ovotestis, was diagnosed in cross-sections as a central lumen, often compressed.

For the analyses presented in Fig. 6, the functional area of a gonad section was defined as the total cross-sectional area minus the area taken up by the oviduct lumen using the ImageJ lasso tool (Schneider et al. 2012). A section’s proportions of testis, ovary, and amorphous/discolored tissue were calculated by dividing their areas by the functional area of the gonad.

To allow the direct comparisons between fish and groups of fish in Fig. 6, we normalized the cross-sectional areas for each tissue type for differences in body size. The normalization factor was found by squaring the ratio of the total body length of a focal fish to the average length of all *K. marmoratus* individuals used in this study. This factor was then applied to the total areas of each tissue type for each fish. All treatment groups were compared by each trait individually using a Kruskal–Wallis rank sum test. This was followed by a Dunn’s post hoc test with *P*-values adjusted using the Benjamini–Hochberg method with a false discovery rate of 0.05 (Benjamini and Hochberg 1995).

## Results

### *Kryptolebias marmoratus* ARs

**Fig. 1:**
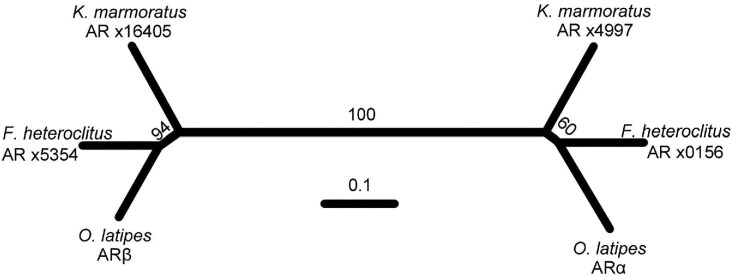
Androgen receptors (ARs) of select Teleosts. Neighbor-joining tree of full-length predicted AR proteins from *K. marmoratus, Fundulus heteroclitus, and Oryzias latipes* (medaka) genomes (Ogino et al. 2009; Whitehead 2009; Kelley et al. 2016). AR-α orthologs form a clade at the right, while AR-β orthologs are at left. Numbers of form “xN” are the final digits of the Ensembl or GenBank gene predictions from which the protein is derived. Numbers at nodes indicate the number of bootstrap subsample replicates (of 100 total) supporting the indicated node.

The latest *K. marmoratus* genome assemblies (Kelley et al. 2016; Snead et al. 2025) contain orthologs of the ARα and ARβ paralogs of medaka and the more closely related cyprinodontiform, *Fundulus heteroclitus* (Fig. 1). Thus, as with other telosts, ARα and ARβ are the likely transducers of androgen signaling in *K. marmoratus.*

### Gonad anatomy of untreated fish

**Fig. 2:**
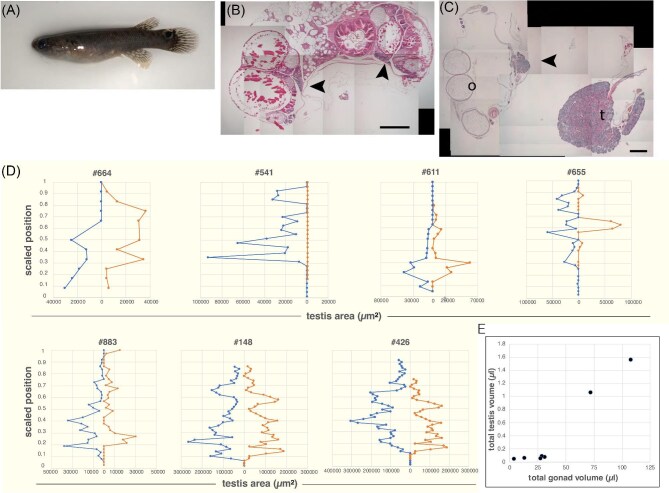
Variation in sex allocation in the *K. marmoratus* hermaphrodite gonad. (**A**) External view of adult hermaphrodite (∼4 cm long), representative of those used for both gonad comparisons and hormone treatments. (**B** and **C**) Sections of gonads from separate hermaphrodites, showing the extreme range of testis content. Scale bar = 1 mm. (**B**) Typicical, largely ovarian section, with small testis pockets adjacent to the oviduct indicated with arrowheads. (**C**) A mostly testicular section, with one gonad (right) completely testis (t), and the other (at left) with a few mature oocytes (o) and a smaller area of testis (arrowhead). This animal appears to be close to completing sex change, yet had outwardly hermaphrodite coloration, similar to “cryptic males” (Marson et al. 2019). (**D**) The absolute area of testis tissue sampled at sections along the transect from anterior (bottom) to posterior (top) in the left and right gonads of seven control (i.e., untreated) hermaphrodites. Specimens are arranged from smallest to largest overall gonad volume. Truncations at the posterior end reflect regions of gonad arm fusion, which are counted in total gonad testis (in F).

Understanding the variation present within untreated control hermaphrodites is necessary to assess the effects of endocrine manipulations. All untreated hermaphrodites examined had both ovarian and testis tissue while maintaining the stereotypical brown hermaphrodite external coloring (Fig. 2A). However, initial specimens quickly revealed that testis tissue was variably abundant, and neither bilaterally symmetric nor uniformly present along the anterior/posterior axis (Fig. 2B and C). We therefore fully reconstructed the gonads of seven hermaphrodites by densely sampling sections along their length (Fig. 2D). This revealed remarkable variation in both overall gonad size and sex allocation. All specimens were between 4.3 and 4.5 cm long, with the expected impact of isometric variation of 14.6% on their gonad volumes. However, the observed volumes varied 25-fold (Fig. 2E). When testis volume was plotted as a function of total gonad volume, a biphasic behavior was seen: Fish with small gonads (<50 µL) invest little in testis (0.2–0.4% of gonad), whereas gonads >50 µL are roughly 1–2% testis.

Beyond total testis endowment, we investigated more find-scaled aspects of testis variation. Bilateral symmetry was generally weak, and one animal (#541) lacked testis entirely in one gonad arm while having substantial amounts in the other. The distribution of testis along the anterior to posterior axis also varied substantially, though there is a significant tendency for an anterior bias (Paired one-tail *t*-test, *P* = 0.041). We note, however, that none of the fish had the complete testis observed in posttransition secondary males with bright orange coloration, which invariably have a complete testis, with no signs of oogenesis or ongoing oocyte atresia (e.g., Fig. 4B).

### Pilot comparison of sex steroids and gonadal sex allocation

The above individual variation in testis content suggested that hermaphrodites may have similarly variable levels of sex steroids, which are produced by somatic gonad cells. To judge whether this was plausible, we used histology and digital image analysis to reconstruct the gonads of three hermaphrodites that had been used as untreated controls in an experiment that appears below (see Fig. 7). We found that testis content and overall gonad volume were weakly correlated with the amount of excreted sex hormones (Fig. S1). Though the sample size is too small to claim significance, it provides additional justification for the hormone manipulations described below.

### Impacts of MT on hermaphrodites

**Fig. 3:**
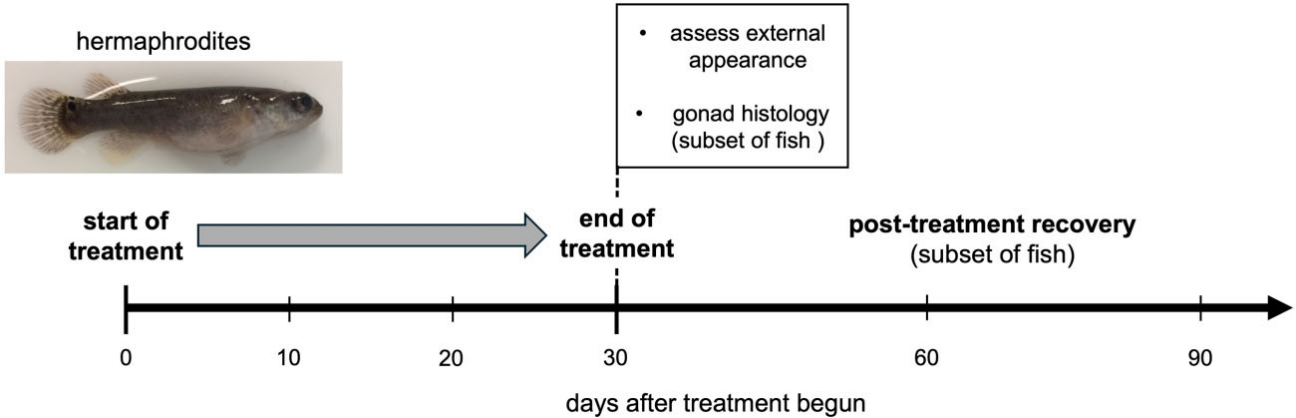
Overview of sex hormone manipulations. Adult hermaphrodites (at least 2 years old) were cultured in water with androgens (MT or KT) or the aromatase inhibitor fadrozole for 30 days, as described in the Methods. Partial water changes to reintroduce drugs occurred at days 10 and 20. After 30 days, fish were removed from drug treatment and either imaged and sacrificed for histological analysis or allowed to recover for at least 60 days.

**Fig. 4:**
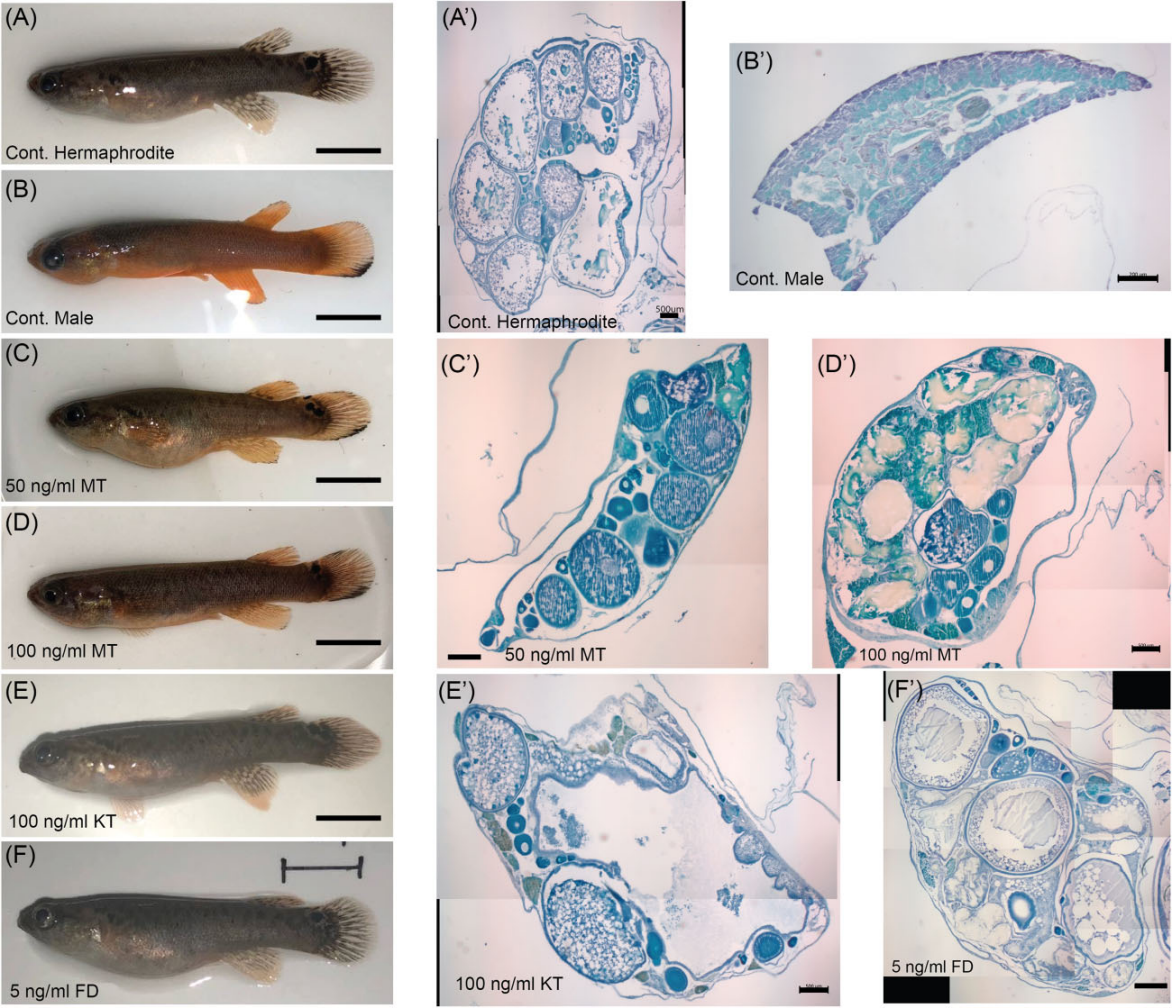
External and internal impacts of exogenous androgen administration. (**A**–**F**) External coloration of fish after 30 days of the indicated treatments. Scale bar = 1 cm. A’–F’: Histological sections of gonads of fish from each treatment, all stained with toluidine blue. Various gonad elements are labeled. T, Testis; EO, early oocytes; LO, late oocytes; AM, amorphous/melanized tissue. Scale bar = 500 µm.

**Fig. 5:**
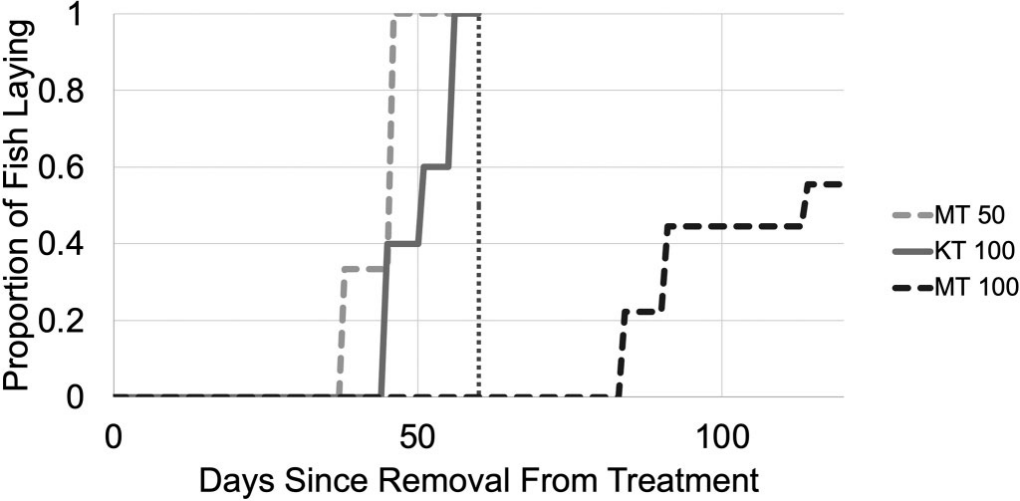
Recovery of self-fertility in post-androgen treatment hermaphrodites. Day zero corresponds to the 30th day of exposure to androgens, after which all fish were removed to fresh water lacking exogenous hormones. The vertical dotted line at day 60 indicates when individuals that had reverted to laying self-fertilized progeny were euthanized. MT 50 (*N* = 9); MT100 (*N* = 9); KT100 (*N* = 5).

As a test of the “androgen snowball” hypothesis, we exposed adult hermaphrodites to different masculinizing endocrine treatments. Impacts of exposure to androgens and fadrozole were assessed as described in Fig. 3. Externally, all 21 MT-treated fish from both the 50 ng/mL and 100 ng/mL groups acquired an orange tint to their coloration, as well as a black bar on their ventral tail fin that is characteristic of males (Fig. 4). The tail bar appeared between the sixth and tenth day of exposure to MT, while the orange tint only started appearing by the fifteenth to twentieth day. Fish treated with 50 ng/mL MT lost their coloration and resumed laying viable self-fertile offspring within thirty days after removal of the hormone. Those treated with 100 ng/mL MT maintained their orange coloration for over 60 days after removal from the hormone, but it faded by day 120, and most began to lay eggs again (Fig. 5). Their tail bars persisted for up to 6 months posttreatment.

**Fig. 6:**
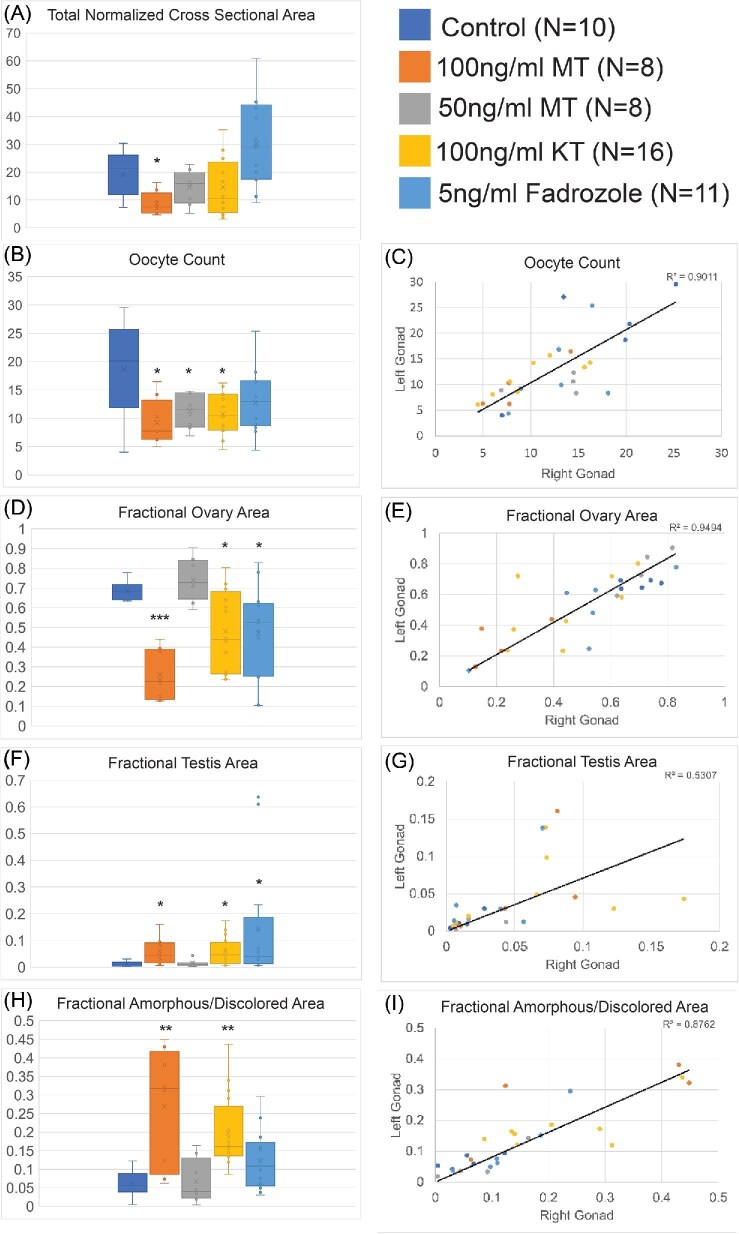
Impacts of hormone treatments on gonad histology. (**A**) Total gonad cross-sectional area, normalized for total fish size. (**B, D, F**, and **H**) Average area per section of gonad arm for the category given. (**C, E, G**, and **I**) Scatter plot of the relationship between the left and right gonad arms within the same individual. Line is a linear regression of all treatments with *R*^2^ value listed in top corner. (**B** and **C**) average oocyte count of every stage between early and late. (**D** and **E**) Percent of gonad volume that is taken up by oocytes. F&G: Percent of gonad volume that is testis. (**H** and **I**) percentage of gonad that is taken up by amorphous/discolored tissue. Reported N is number of gonad arms measured per treatment. The odd number of gonads for the fadrozole treatment resulted from a partially damaged specimen (not used in panels C, E, G, and I). Significance of differences in values for each treatment as compared to control (two-tailed *T*-test): *<0.05; **<0.005; ***<0.0005. Each box-whisker displays the mean (X) and median (horizontal gray line) values, with the middle two quartiles boxed and extreme low and high values indicated by whisker termini.

Internally, the gonads of fish treated with 100 ng/mL of MT showed a range of effects. There was a significant decrease in normalized cross-sectional area, ovary percentage, and oocyte number, while both testis and amorphous/melanized tissue took up a significantly higher proportion of the gonadal volume compared to controls (Fig. 6). Based on studies of other teleost fish, the pigmented tissue likely indicates the presence of melano-macrophages (Kumar and Joy 2015) and associated oocyte atresia (Blazer 2002). Despite its ability to induce external pigment changes, 50 ng/mL of MT did not significantly change any gonad trait other than oocyte count versus the controls.

### Impacts of KT and fadrozole on hermaphrodites

As MT treatments failed to trigger irreversible sex change, we conducted follow-up studies with the natural androgen, KT, and the aromatase inhibitor fadrozole. The ability of fadrozole to inhibit aromatase-mediated production of estrogens suggested that its introduction could simultaneously promote androgen accumulation and inhibit estrogen production. Such a coordinated shift in both sex-steroid classes could more closely mimic natural sex change. However, neither exogenous KT nor fadrozole triggered any noticeable external coloration change in the fish during or after exposure (Fig. 4). Fish treated with exogenous KT did have significantly less ovarian content and more testis and amorphous/discolored tissue content in their gonads than controls, but overall gonad size was not affected (Fig. 6). Fadrozole at 5 ng/mL increased the fraction of testis tissue, but caused no changes in relative gonad size or in ovarian or amorphous tissue content (Fig. 6). Overall, the KT and fadrozole treatments induced gonad-specific masculinization that was weaker than that of the 100 ng/mL MT treatment.

**Fig. 7:**
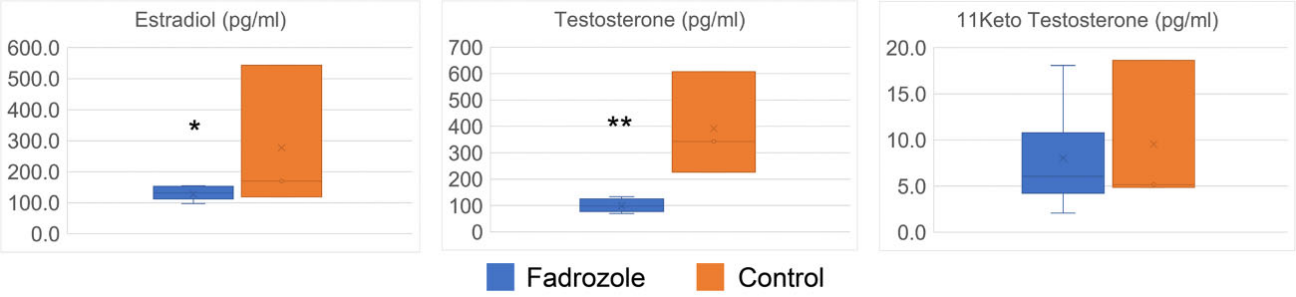
Impact of aromatase inhibition on sex steroids. Terminal hermaphrodites treated with 5 ng/mL of fadrozole (*N* = 6) or untreated control individuals (*N* = 3, same individuals as in Fig. 2F) were compared. Reported estradiol, testosterone, and 11-ketotestosterone concentrations are for resuspended concentrates (see Methods for details). *P*-values *<0.05; **<0.005

Because it was not clear how the sex steroid profile of a simultaneous hermaphrodite would react to aromatase inhibitor, we sought to measure it directly using hormone immunoassays. Fadrozole exposure led to a decrease in both endogenous testosterone and estradiol in the fish, while KT was not changed (Fig. 7).

## Discussion

### Stochastic hermaphrodite testis development

Kanamori et al. (2006) reported that testis cysts could be observed in some juvenile hermaphrodite *K. marmoratus* gonads as early as two months post-hatch, and were reliably present 82 days post-hatch. The earliest testis rudiments were not symmetrical between left and right gonad arms, and appeared in an anterior-biased distribution. We sought to extend this work to self-fertile adults, which all have at least some testis tissue. In particular, we wondered if the within- and between-individual variation observed by Kanamori et al. (2006) persisted into adulthood. Our results clearly demonstrate that it does. The degree of individual asymmetry can be striking, with the gonad arms of one specimen essentially being one ovary and one testis (Fig. 2c). It is noteworthy that even such a testis-rich individual still appeared hermaphroditic externally. Field-caught *K. marmoratus* with complete bilateral testes have also been reported to show similar hermaphrodite-like external pigmentation, though subtle differences are often present (Marson et al. 2019). Whether these cryptic males are a stable alternative reproductive mode or a transient form that precedes the appearance of overt male pigmentation is unclear. If transient, this implies that external coloration is the last male trait to appear during a normal sex change.

### Inadequacy of the “androgen snowball” hypothesis

The variation of testis content seen in *K. marmoratus* hermaphrodites (and potentially the endogenous androgen levels this may dictate) mirrors the similarly variable timing of sex change in even genetically identical fish reared in a common environment (Davis et al. 1990; Turner et al. 2006). If the sensitivity of testis development to elevated androgen levels seen in very young animals (Kanamori et al. 2006) persisted into adulthood, this could cause positive feedback between androgen levels and masculinization of the gonad to drive sex change. However, in subsequent experiments, a simple positive feedback model was not supported.

Because adult fish have a smaller ratio of surface to volume than larvae, and may metabolize exogenous hormones differently, we substantially increased both the dose and length of treatment relative to Kanamori et al. (2006). The highest dosage of MT given in our experiments (100 ng/mL) was four times higher, and the duration of exposure (30 days) was three times as long. External pigmentation of these 2 + year-old hermaphrodites did become male-like in both MT treatments (Fig. 4), and there were signs of masculinization in the gonad (Fig. 6), indicating our doses were effective. However, even the high dose of MT was unable to trigger a complete transition of the gonad to testis. Furthermore, treated pseudo-males lost male pigmentation and transitioned back to self-fertility after removal from exogenous hormones (Fig. 5). Our lower MT dose, 50 ng/mL, was also capable of triggering male coloration externally. However, while still twice the level sufficient for juvenile masculinization, it induced almost no signs of gonad masculinization, with a drop in oocyte count being the only significant change (Fig. 6). While longer-term MT treatments may be informative, our 30-day treatments were longer than the transition time from egg-laying hermaphrodite to orange male observed in our *K. marmoratus* colony, which is typically less than 2 weeks.

The incomplete and reversible masculinization observed with MT led us to look at other androgens and hormone modifiers. KT was an obvious next choice, as it has high activity in teleost fish (Borg 1994). Disrupting aromatase activity with fadrozole had the potential to both lower estrogen and increase androgen levels. Further, both KT and fadrozole can masculinize other teleosts (Bhandari et al. 2004; Kroon et al. 2005). However, while both treatments showed some facets of masculinization in the gonad, they also failed to fully masculinize any individual (Figs. 5 and 6).

While *K. marmoratus* and other teleosts have two orthologous ARs (Ogino et al. 2009; Fig. 1), they can mediate divergent roles of androgens in development (Ogino et al. 2018). Their expression in the gonads of Japanese eels (Todo et al. 1999) and mouse knockouts (Yeh et al. 2002; Chang et al. 2004; De Gendt et al. 2004; Holdcraft and Braun 2004) suggest a conserved role in gonadal sex. Consistent with this, zebrafish ARs are necessary for normal testis development and spermatogenesis (Tang et al. 2018). Surprisingly, however, this requirement is not true of all teleosts. In medaka (*Oryzias latipes*), XY fish harboring single and double AR knockouts have normal male gonads and sperm. Though these receptors seem to have lost their role in testis development and spermatogenesis, they each have important, and different, roles in male behaviors and morphology (Ogino et al. 2023). In *K. marmoratus*, a similar subfunctionalization may explain the differences in coloration, with KT reacting strongly with one receptor and not the other.

With the above taxonomic variability in mind, it is notable that MT is sufficient to masculinize juveniles (Kanamori et al. 2006), yet insufficient to irreversibly masculinize adult hermaphrodites, even at higher doses and longer exposures. *Kryptolebias marmoratus* ARs thus appear to have a role in early testis specification that is similar to mammals and reptiles. Whether androgens act on the gonad directly or indirectly (e.g., via pituitary gonadotropins), they can no longer effect such a complete masculinization in adults with established ovotestes. The difference in effects of KT and MT on external pigmentation is also puzzling, as both androgens bind ARs, and KT is thought to be the most consistently male-associated androgen in many teleosts (Borg 1994). One likely explanation for the heterogeneous responses across the body may be variation in the expression of the ARs themselves, which could differentially sensitize different tissues. This is an important avenue for future research.

Our results are inconsistent with the hypothesis of androgen-driven positive feedback being a core feature of the natural sex change mechanism. It is possible that hormonal perturbations could fully masculinize with even higher doses or longer exposure times. However, given that substantial shifts toward the testis fail to initiate a full sex change, it appears an unknown signal upstream of gonad sex is needed to allow complete masculinization. One fruitful avenue for future investigation would be to compare the expression of AR paralogs in hermaphrodite and male *K. marmoratus.* Elevated expression in the male brain and/or gonad may be a prerequisite for sex change, sensitizing tissues and enabling the sort of feedback we have envisioned here. Whatever the underlying trigger for sex change is, alternative configurations of it presumably underlie the variation of sex change timing and male frequency within *K. marmoratus* strains (Sakakura and Noakes 2000; Mackiewicz et al. 2006c; Costa 2016; Gresham et al. 2025).

### Response to fadrozole

Fadrozole treatment was unable to trigger external pigment changes or loss of oocytes (Fig. 4), though it did moderately stimulate testis growth (Fig. 6). To better understand the impact of this drug on steroid titers, we measured testosterone, estradiol, and KT secretion in control and fadrozole-treated animals. As has been previously reported (Garcia et al. 2016), hermaphrodites produce testosterone in amounts comparable to males, despite their largely female anatomy. KT and estradiol levels are both elevated roughly twofold in males, the latter being unusual for a teleost. Surprisingly, while fadrozole reduced secreted estradiol levels, as expected from aromatase inhibition, it also led to an unexpected simultaneous drop in secreted testosterone. In addition, it did not significantly reduce KT levels, even though KT can be synthesized directly from testosterone. Thus, fadrozole exposure did not shift secreted sex steroids in a consistently masculinizing direction, as we had expected. It is thus perhaps not surprising that no masculinization of external pigmentation and only weak masculinization of the gonad were observed. Our noninvasive hormone assay is indirect, and may not precisely reflect serum steroids, but the large shifts observed in testosterone and estradiol are likely qualitatively robust to this.

Reduction of testosterone by fadrozole is surprising, as other teleost fish have been reported to respond by either having no change (Afonso et al. 1999) or an increase (Ankley et al. 2002; Huffman et al. 2013). The response in *K. marmoratus* hermaphrodites may reflect a common origin in tissues that were impacted by hormone treatment (e.g., ovary), a biosynthetic feedback loop that represses testosterone when estrogen production is interrupted, or some other mechanism. Importantly, our assays cannot determine a hormone’s tissue of origin. Though we have emphasized gonadal steroid production here, the brain is also a source that can have nonintuitive functions. For example, brain-derived estrogen plays an essential role in male behaviors in medaka (Nishiike et al. 2026). Given the sex-reversed levels of estradiol measured by Garcia et al. (2016), this may be relevant to *K. marmoratus* as well.

### Independent trajectories of gonadal sex change

When monitoring testis development after hormone-mediated masculinization, we observed a point where either the left or right gonad develops testis at a rapid rate, while the other maintains a primarily ovarian character (Fig. 6G). The percentage of variance in the testis content of one gonad that is explained by that of the other (R^2^ value) is dramatically lower than that of other metrics, especially for the fish with elevated testis content. This indicates each gonad behaves somewhat independently as masculinization progresses. Consistent with this, one overtly hermaphroditic control fish examined had a complete testis on one side, and an ovo-testis on the other (Fig. 2C), while in another one arm lacked testis entirely (Fig. 2D).

### Gonad and external phenotype discordance

The de-synchronization of gonad sex allocation and external coloration seen in the fadrozole and KT-treated individuals may be relevant to how *K. marmoratus* males naturally develop. Across years of research in the Haag Lab and others, unmanipulated individuals with male coloration always have complete bilateral testes, consistent with more systematic reports (Harrington 1971; Marson et al. 2019). However, 50 ng/mL MT-treated fish had masculine coloration, yet only very weak gonadal masculinization. Conversely, both KT- and fadrozole-treated fish were partially masculinized in their gonads but had no external coloration change (Fig. 4). These results suggest that during natural sex change, circulating testosterone needs to reach a threshold level for coloration change that is only attained with complete gonad masculinization. Application of exogenous MT via immersion achieves this independent of gonadal contributions. The discovery of cryptic male *K marmoratus* (Marson et al. 2019) is consistent with this model of male coloration as an androgen-mediated secondary effect of gonad physiology.

The ability of exogenous MT to alter pigmentation without similar masculinization of the gonad could, in principle, reflect direct action of the hormone on ARs in skin pigment cells that is independent of the circulation or the endogenous brain-gonad axis. However, gonad changes confirm our hormone treatments penetrated the skin, and similar masculinization of external coloration in the absence of ovarian disruption was observed in bitterling females treated with MT and KT via feeding (Kobayashi et al. 2009). This suggests the gonad may be intrinsically less sensitive to circulating androgens than are skin chromatophores in some species. It is also possible that our histological assays simply fail to capture gonad changes that drive pigment masculinization. The inability of KT to mimic MT’s effects on pigmentation in our experiments (even though KT modestly masculinized the gonad) differs from the results of Kobayashi et al. (2009) in bitterling. In *K. marmoratus* these two androgens elicit distinct responses, despite indications they bind the same ARs and that KT is the more potent agonist (Olsson et al. 2005). One possible explanation is differential metabolism of the two androgens, with KT perhaps being more labile and the artificial MT more persistent.

### Tentative model of *K. marmoratus* sex change

Social interactions in *K. marmoratus* can regulate endorocrine states and behaviors (e.g., Earley and Hsu 2008; Garcia et al. 2015). These may influence sex allocation in field conditions. However, fish reared in isolation in the laboratory change sex in the absence of such interactions, and it is this phenomenon we are best positioned to address. Based on our results and those of others, we propose a tentative model for the order of events in the spontaneous transition of *K. marmoratus* hermaphrodites into males:

Step I: Transition commitment signal. An unknown factor commits a fish to begin the transition process. As sibling fish from inbred lines reared in isolation still vary, this signal is unlikely to be genetic or social. The pioneering experiments on sex change in *K. marmoratus* by Harrington (1971) suggested one trigger may be short day length. Though this may be true in the Florida populations he studied, our fish are kept on a constant 12–12 light cycle, yet change sex anyway at an appreciable rate. Further, some *K. marmoratus* populations are near-equatorial, so that day length variation is minimal. This suggests that there must be other innate triggers of sex change that remain to be discovered. However, it cannot be replaced by exogenous androgens, nor by the modest elevation of testis content within the gonad they induce.
Levels of cortisol or of other hormones in the brain may change with age, and in another protogynous fish, the blue-headed wrasse, sex change though to be regulated by changes in stress and cortisol levels (Solomon-Lane et al. 2013). Removal of the dominant male reduces cortisol in larger females, in which transition is de-repressed. Though *K. marmoratus* sex change is not tied to social interactions, measuring cortisol levels or other factors along the sex steroidogenic pathway throughout their lives might still be able to predict sex change. Possible sources of stress may include environmental factors. However, all fish were kept in a 12–12 light–dark cycle in a small, common room and received equal feeding, yet we still see variability in sex change among individuals from highly inbred lineages.
Step IIa: Gonad masculinization. As shown in Fig. 2, testis development can occur on rather independent timetables in each gonad of an individual and to a great extent in the absence of external pigmentation changes. Even animals with essentially complete testes can retain a hermaphrodite external appearance (Marson et al. 2019).
Step IIb: Oocyte atresia. Both doses of MT induced significant reductions in oocyte number and ovarian area, and significant increases in amorphous and/or melanized tissue. This is similar to the effects of injected MT (Park et al. 2013), and explains the significant drop in total gonad area in the high MT dose. While similar atresia is necessary to eliminate ovarian tissue in proto-males as they transition, elevated androgens may not be driving this process in spontaneous sex changes. Melanized ovarian tissue during oocyte atresia (resorption) has been described in other fish, and is associated with melanomacrophage infiltration (Kumar and Joy 2015).
Step III: Secondary Secondary Sex Characteristics. After gonad remodeling is complete, endogenous androgen levels rise to levels sufficient to trigger formation of the ventral caudal fin tail bar, fading of the caudal ocellus, and concentration of carotenoids in the posterior skin. Our treatments were sufficient to mimic this independent of endogenous gonadal steroids. However, male pigmentation faded when exogenous modulators were withdrawn, likely due to the inability of exogenous hormones to trigger complete the gonadal masculinization that would sustain male appearance long-term.

## Conclusion

The unpredictable timing of sequential hermaphroditic transition, even between genetically identical individuals, is mirrored in the variable gonadal sex allocation between individuals and even between the left and right gonad arms of the same individual. All of this makes *K. marmoratus* “consistently inconsistent” in its sexual characteristics. Gonadal sex allocation in *K. marmoratus* can be influenced by hormone perturbations, but this alone is insufficient to allow the complete transformation from hermaphrodite to male. This indicates the Androgen Snowball Hypothesis is inadequate and that other factors must be at play for sex change to occur. Further exploration should focus on upstream (i.e., brain and pituitary) regulators of gonad development and both sexual and stress-related steroid signaling pathways. One possible approach would be to compare GnRH and LH levels between juveniles of typical strains that form males rarely and slowly with those that form males early and often, as in Twin Cays, Belize (Mackiewicz et al. 2006c; Turner et al. 2006).

## Supplementary Material

obag009_Supplemental_File

## Data Availability

Data used to produce Fig. 1 are available from ENSEMBL (www.ensembl.org) as described in the Methods. Data underlying Figs. 2, 4, 6, and 7 are available upon request.
