# Supplementary material for: Resistance of Adult Kryptolebias marmoratus Hermaphrodites to Irreversible Sex Change by Exogenous Androgens
Source: Integr Org Biol. 2026 Mar 11;8(1):obag009. doi: 10.1093/iob/obag009 (PMC13032168; doi:10.1093/iob/obag009)
Supplement: obag009_Supplemental_File [file obag009_supplemental_file.pdf]

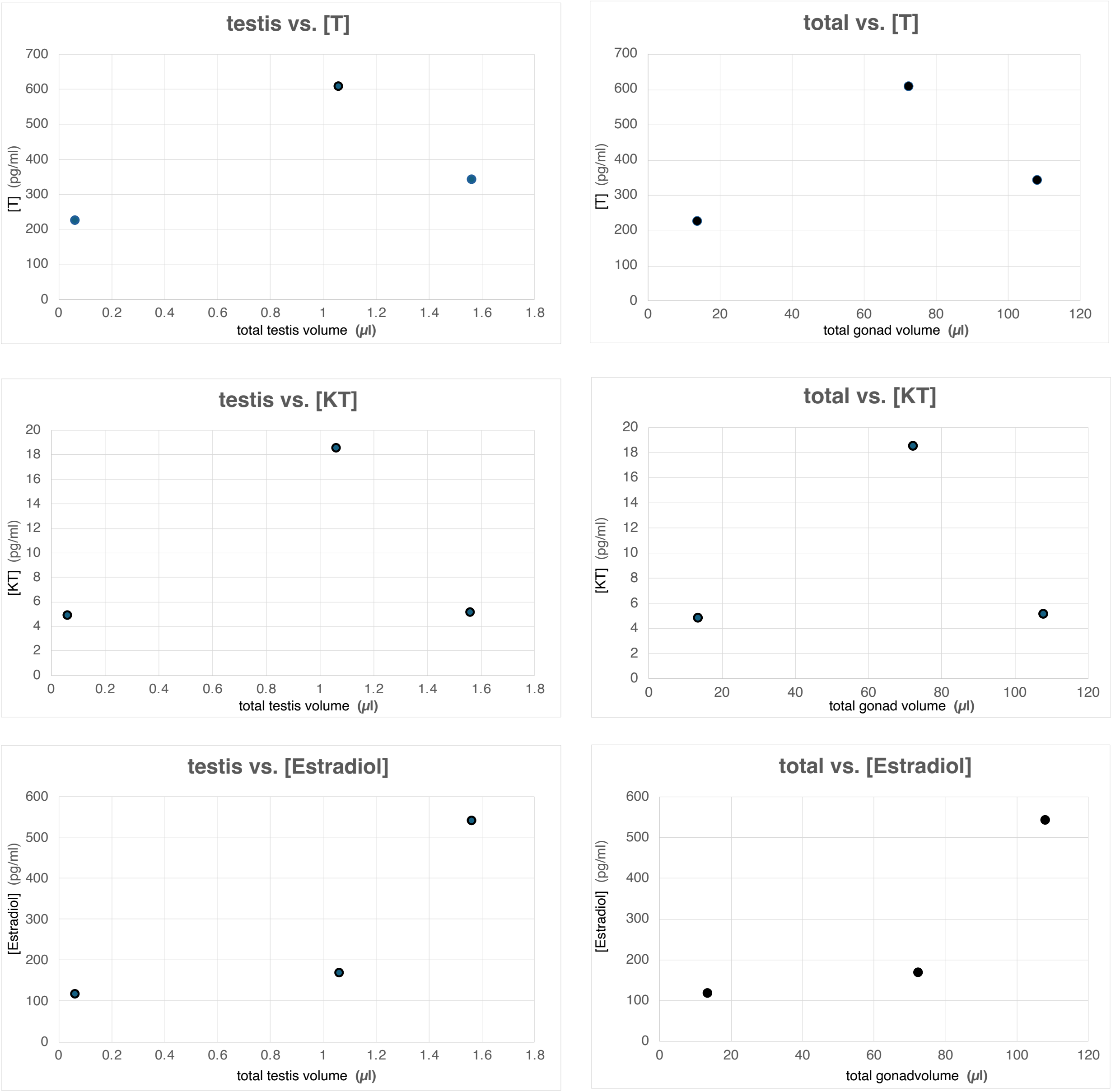


**Supplemental Figure S1**: Pilot study of relation between testis and total gonad volume and excreted sex steroids (same samples as untreated controls in Fig. 7). No correlations are significant at P=0.05. The hypothesized correlation between testis content and androgens are 0.47 for testosterone and 0.16 for 11-ketotestosterone. The correlations between testis and estradiol and between total gonad volume and estradiol were both 0.85.

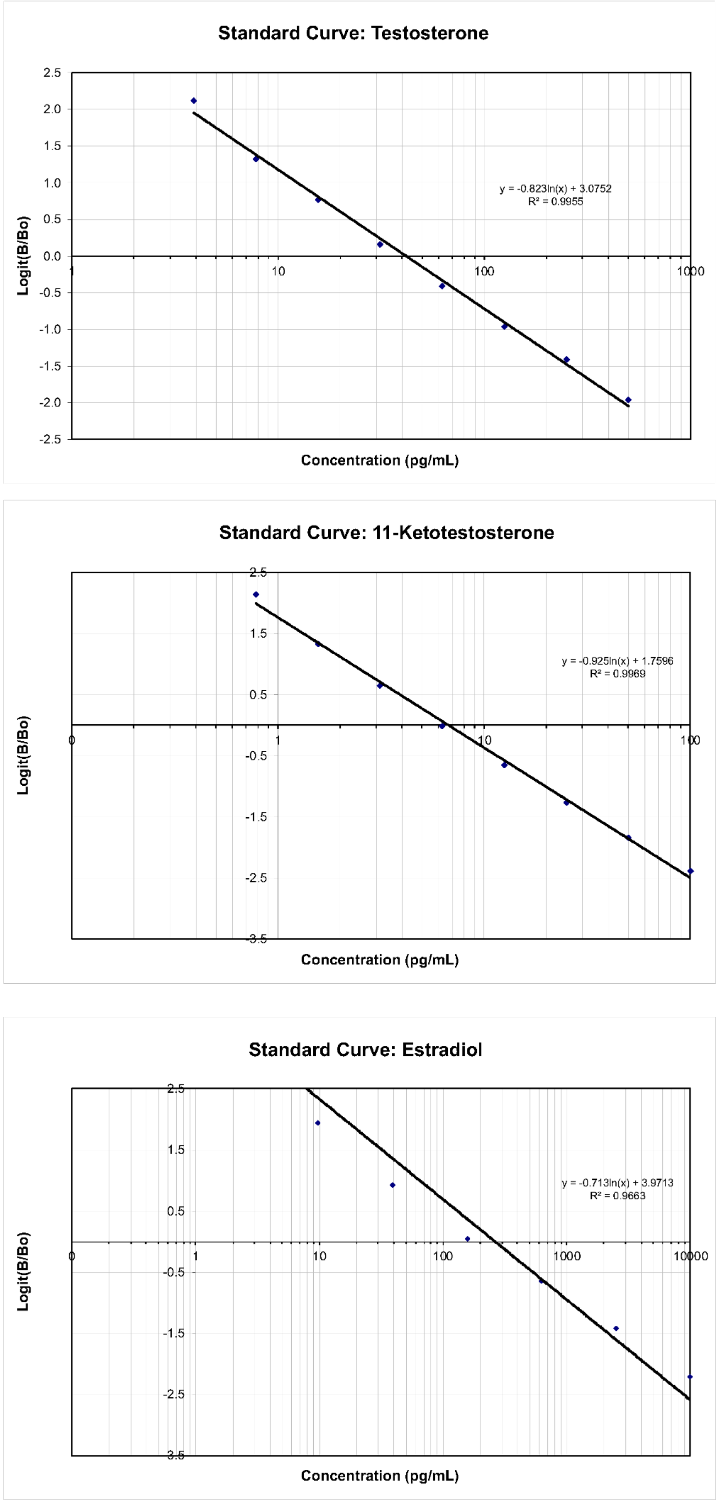


**Supplemental Figure S2: Hormone ELISA assay standard curves.** Hormone standards were serially diluted in two-fold steps. The slope of the log-linear plot and fraction of variance explained by the dilution process ($R^2$) are indicated on each plot. Unknown samples quantified in Figure 6 all fall within the range of each standard curve.
